# Application of the decision tree method to lithology identification of volcanic rocks-taking the Mesozoic in the Laizhouwan Sag as an example

**DOI:** 10.1038/s41598-020-76303-y

**Published:** 2020-11-05

**Authors:** Yajun Duan, Jun Xie, Yanchun Su, Huizhen Liang, Xiao Hu, Qizhen Wang, Zhiping Pan

**Affiliations:** 1 College of Earth Science and Engineering, Shandong University of Science and Technology, Qingdao, 266590 Shandong China; 2 CNOOC China Limited, Tianjin Branch, Tianjin, 300459 China; 3 College of Mechanical and Electronic Engineering, Shandong University of Science and Technology, Qingdao, 266590 Shandong China

**Keywords:** Geology, Petrology, Volcanology

## Abstract

The decision tree method can be used to identify complex volcanic rock lithology by dividing lithology sample data layer by layer and establishing a tree structure classification model. Mesozoic volcanic strata are widely developed in the Bohai Bay Basin, the rock types are complex and diverse, and the logging response is irregular. Taking the D oilfield of the Laizhouwan Sag in the Bohai Bay Basin as an example, this study selects volcanic rocks with good development scales and single-layer thicknesses of more than 0.2 m as samples. Based on a comparison of various lithology identification methods and both coring and logging data, using the decision tree analysis method and the probability density characteristics of logging parameters, six logging parameters with good sensitivity to the response of the volcanic rocks of the above formation are selected (resistivity (RD), spontaneous potential (SP), density (ZDEN), natural gamma ray (GR), acoustic (DT), and compensated neutron correction (CNCF) curves), which are combined to form a lithology classifier with a tree structure similar to a flow chart. This method can clearly express the process and result of identifying volcanic rock lithology with each logging curve. Additionally, crossplots and imaging logging are used to identify the volcanic rock structure, and the core data are used to correct the identified lithology. A combination of conventional logging, imaging logging and the decision tree method is proposed to identify volcanic rock lithology, which substantially improves the accuracy of rock identification.

## Introduction

Volcanic rock, as an important basin filler, is the main component of the early basin formation period. It is believed that all volcanic rocks may become reservoirs. The composition and structure of volcanic rocks are complex. There are many types of rocks and unclear logging relationships, making lithology identification difficult. However, the identification of volcanic lithology is the basis for the division of volcanic lithofacies and the study of reservoir performance and characteristics, which is significant to production practices. Therefore, it is necessary to study the lithology identification method of complex volcanic rocks^1–4^. At present, there are four main methods for volcanic rock lithology identification. The first method is to use core, thin-section and logging data to identify lithology directly and effectively. However, because volcanic rocks mostly develop at the bottom of a basin, drilling and coring are difficult, and it is difficult to apply this method of identification to an entire well section. The second method is to identify the lithology of volcanic rocks by seismic or gravity-magnetic-electric methods, which mainly use the seismic reflection characteristics of different lithologies of volcanic rocks and the influence of volcanic rocks on the magnetic anomaly intensity to identify lithology^5^ This kind of method can only identify the macroscopic distribution range of volcanic rocks and not the detailed identification of the volcanic rock lithology^6,7^. The third method is to use special logging data, such as element logging and imaging logging, for lithology identification^8–10^. This method has a good identification accuracy but a high cost, and most old wells lack special logging data, making it difficult to apply this identification method to all wells in a certain area^11,12^. The fourth method uses a conventional logging curve to identify volcanic rock lithology; commonly used methods include the conventional logging curve feature method, crossplot identification method, principal component analysis method and neural network method^13–17^. This method is the most commonly used for volcanic rock lithology identification.

During the formation process of the Bohai Bay Basin, it experienced two rifting cycles in the Mesozoic and Cenozoic^18–20^, forming numerous intermediate acid and intermediate basic volcanic rock formations^21–23^. The D oilfield is located in the Laizhouwan Sag in the southeast of the Bohai Bay Basin. Drilling investigations have shown that volcanic reservoirs in this area have great development potential^24,25^. However, due to the complexity of the volcanic eruption environment, frequent eruption cycles and periods, lithology diversity and different degrees of weathering and alteration during diagenesis, the complex lithology of volcanic rock, high exploration cost of offshore platforms, limited data collection and difficult lithology identification restrict further exploration of volcanic reservoirs; thus, it is urgent to determine an effective approach for identifying volcanic rock lithology^26,27^. To solve the complex problem of lithology identification of volcanic rocks, the decision tree method of the "white box" model is introduced. The logging curves are screened layer by layer to clearly describe the various elements, related factors and process rules in the process of logging curve identification^28^. The tree structure lithology classifier is established to distinguish complex lithology, effectively solving the problem of the low accuracy of lithologic profile identification caused by the lack of large-scale and continuous cores. On the basis of selecting six logging parameters for lithology classification, identification and screening, this study uses the decision tree method to first generally distinguish lithology, which is corrected with imaging logging identification and core identification, and ultimately identify the volcanic rock lithology in the study area in detail.

## Geological background

**Figure 1:**
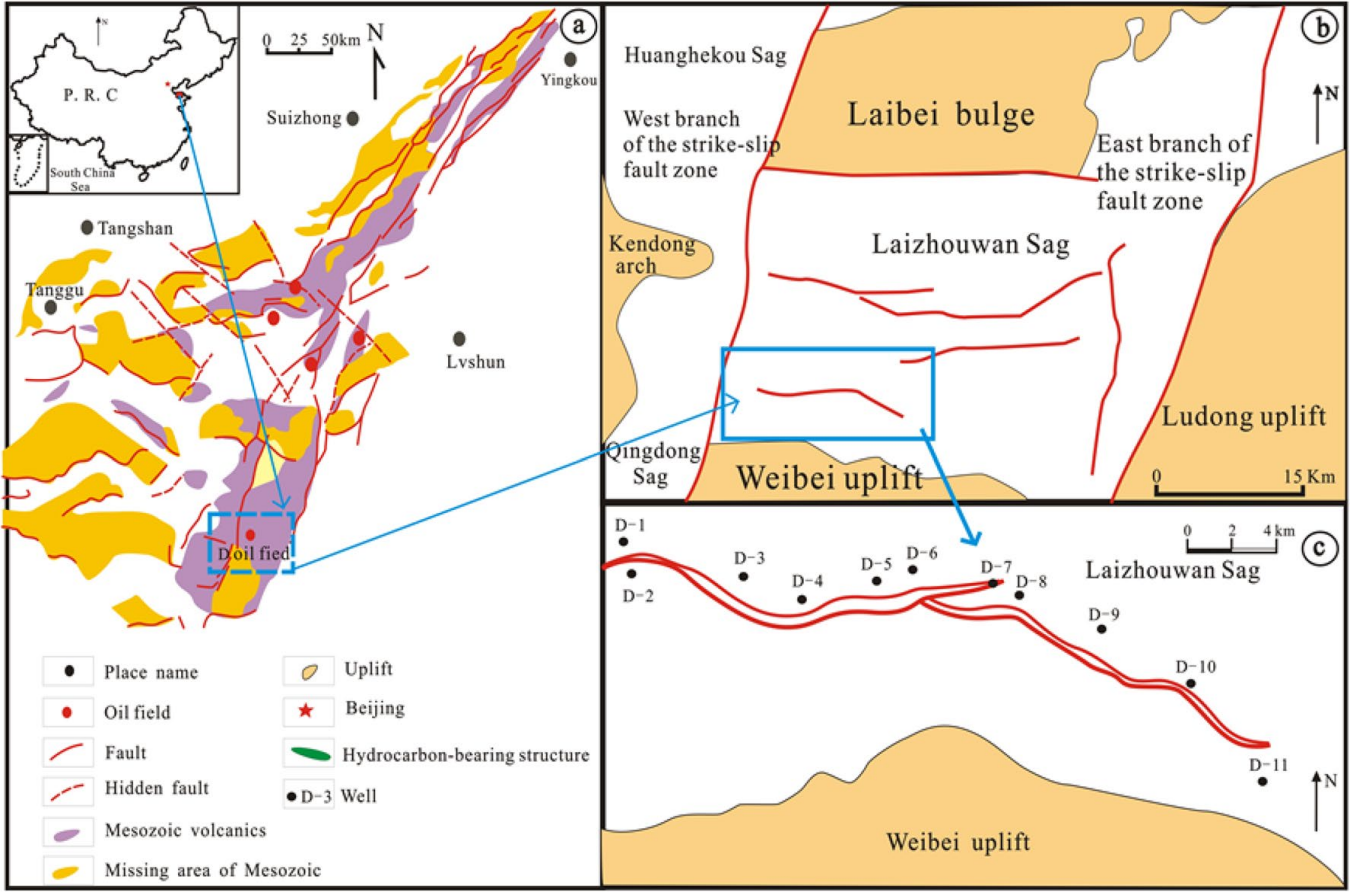
Distribution of major oil and gas fields in the Kenli area and location of the Kenli area. Map generated using CorelDRAW 2017. (https://www.corel.com/) (**a**) Laizhouwan Sag location map. (**b**) D oilfield location map. (**c**) D oilfield well location schematic.

**Figure 2:**
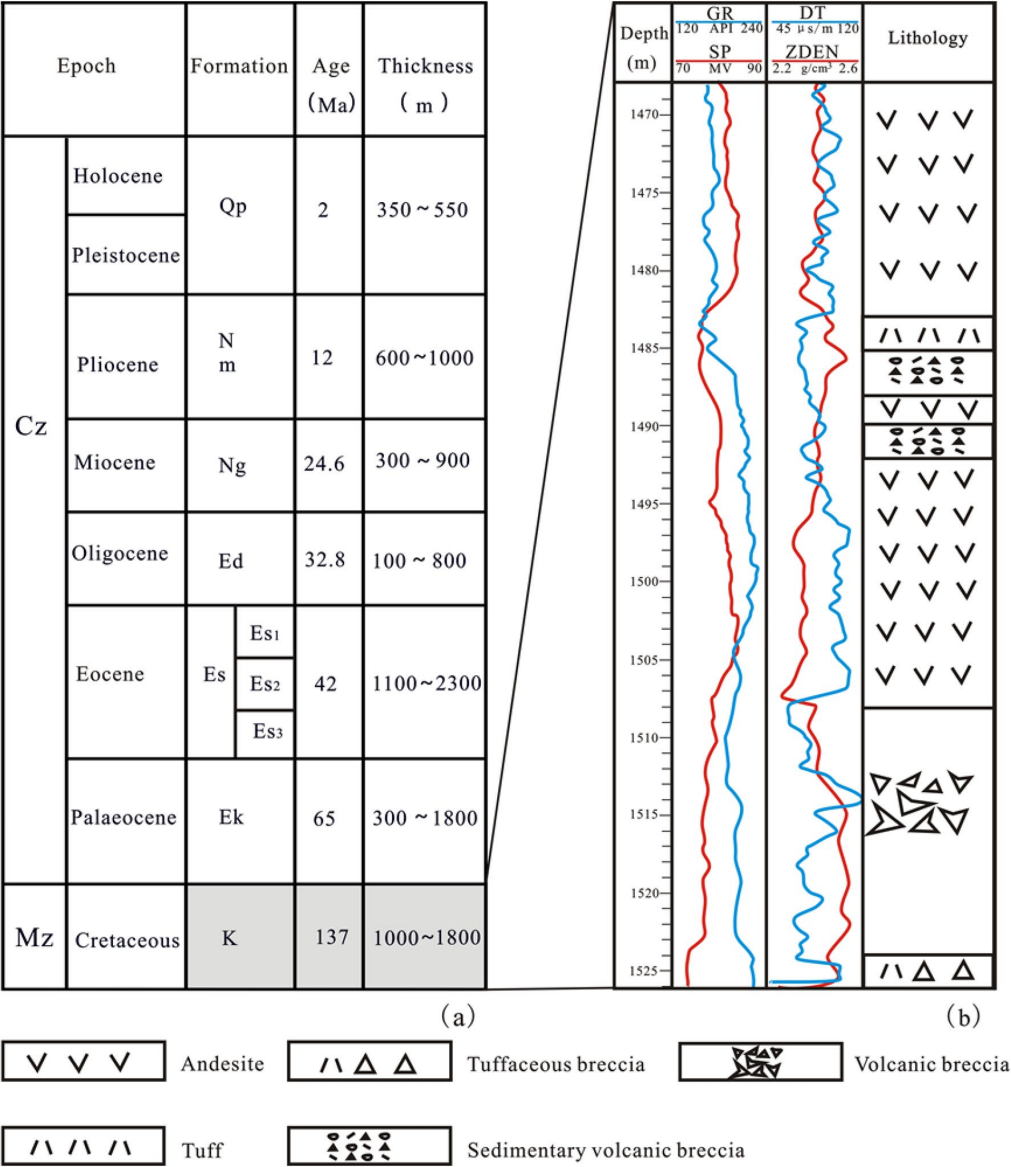
(**a**) Lithostratigraphy of the Laizhouwan Sag. (**b**) A detailed stratigraphic column of well D-6.

The Laizhouwan Sag, located in the southeastern Bohai Bay Basin, is a Cenozoic depression developed on a Mesozoic base^29–31^ (Fig. 1a). Strong volcanic activity occurred during Mesozoic rifting. The D oilfield is located in the southern slope zone of the Laizhouwan Sag (Fig. 1b,c). The buried Mesozoic hill in the study area is located at a high position, close to the hydrocarbon generation centre of the Laizhouwan Sag, resulting in the good trap conditions, well-developed fractures and good reservoir-forming conditions of the volcanic reservoir. Well drilling investigations have revealed that the Neogene Guantao Formation, Palaeogene Shahejie Formation and Mesozoic strata all have strong oil and gas shows^32^. This area is in a low-exploration area and has great exploration potential^33^. Among these strata, the Mesozoic mainly developed the Cretaceous Yixian Formation and Jurassic Lanqi Formation volcanic strata with complex lithologies, which are the main target strata of this study^34–36^ (Fig. 2).

## Samples and methods

A total of 235 core samples, with diameters of 50 mm and heights of 80 mm, were collected from the ten cored wells in the core library of the D oilfield. A total of eight logging curves were collected for each well. Among them, imaging logging curves were collected from four wellbores. The lithology identification of volcanic rocks in the study area mainly adopted the slice identification method, intersection graph method, decision tree method and imaging logging method. The analysis method is summarized as follows.

### Slice observation

The rock was cut to a thickness less than 0.3 mm and placed under a slide with adhesive to observe the mineral composition of the rock. Out of the 235 samples in the study area, 137 samples were identified by polarizing microscopy. The remaining 98 samples were made into thin casting sheets. The samples for preparing thin sections were impregnated with alizarin epoxy resin and potassium ferricyanide under vacuum to clearly observe the pores^28^. Dickson's method was used to distinguish pores from grains.

### Intersection graph method

The intersection of the resistivity curve (RD), density curve (ZDEN), compensated neutron correction curve (CNCF) and natural gamma ray curve (GR) was selected for detailed lithology identification of volcanic rocks to obtain the approximate distribution range of the logging response parameters corresponding to different lithologies^37,38^, which is the basis of the decision tree method.

### Imaging logging method

Imaging logging uses the electrical conductivity of a formation to convert the microresistivity curve into bright and dark spots to reflect the lithologic characteristics and fracture conditions of the formation. The higher the formation conductivity and the lower the resistivity are, the darker the colour will be^39,40^. Using Fullbore Formation MicroImaging (FMI) technology, four wells were imaged to reflect the stratigraphic features visually, and these results were combined with conventional logging data for lithology identification in the study area.

### Principle of the decision tree method

A decision tree is a tree structure similar to a flow chart^28,41,42^. Its principle is to test different data samples, divide samples with different results into different sample subsets, and finally establish a tree structure model, for which each branch represents the output of a test point that can determine the relationship between data sample records and sample attributes^43^. Through the classification and prediction of logging data samples, the relationship between various logging curves and the volcanic rock lithology can be clearly described. Therefore, based on the interactive graph method, the decision tree model is used to segment the lithology, which decreases the information entropy of each subset and lithology type and finally generates a classification decision tree for lithology identification.

## Result

### Recognition of lithologic slices of volcanic rocks

According to the core thin-section data of each well in the study area, the core observation data and the borehole wall data, the main types of Mesozoic volcanic rocks in the study area are volcanic clastic rock and volcanic lava, and the volcanic clastic rock includes normal volcanic clastic rock and sedimentary pyroclastic rock. A small amount of pyroclastic sedimentary rock, mainly tuffaceous conglomerate, can be seen in the lower part of wells D-7 and D-10; andesite, rhyolite and dacite can be observed in the volcanic lava.

**Figure 3:**
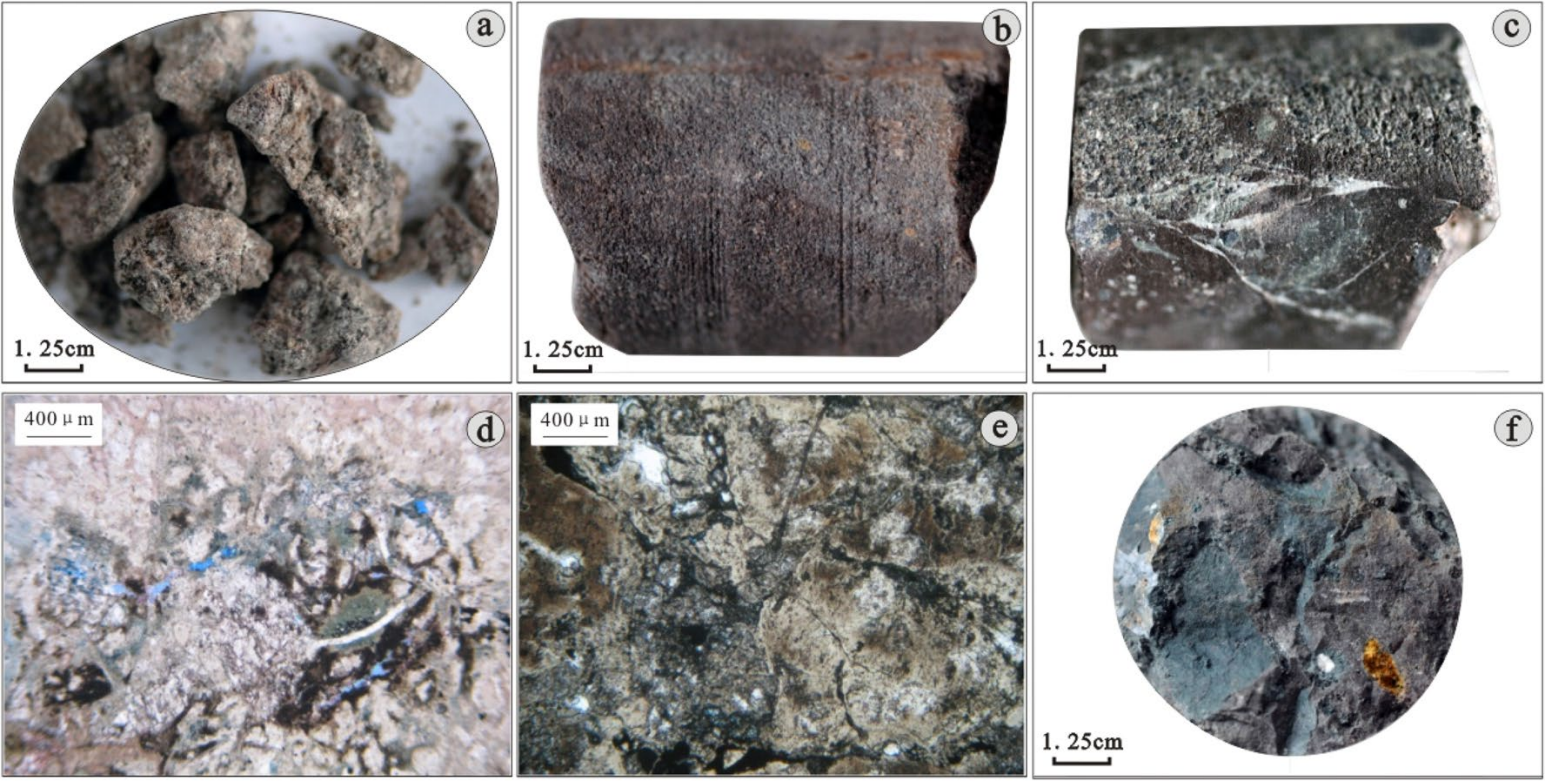
Photographs of the characteristics of Mesozoic volcanic clastic rocks in the D Oilfield, Laizhouwan Sag. (**a**) Tuff D-4 1203.5 m massive structure. (**b**) Tuff D-7 1588 m purple brown. (**c**) Volcanic breccia D-7 1636 m variegated. (**d**) Volcanic breccia D-9 1605 m. (**e**) Brecciferous rhytitic tuff D-9 1603.68 m. (**f**) Tuffaceous breccia D-7 1580 m.

**Figure 4:**
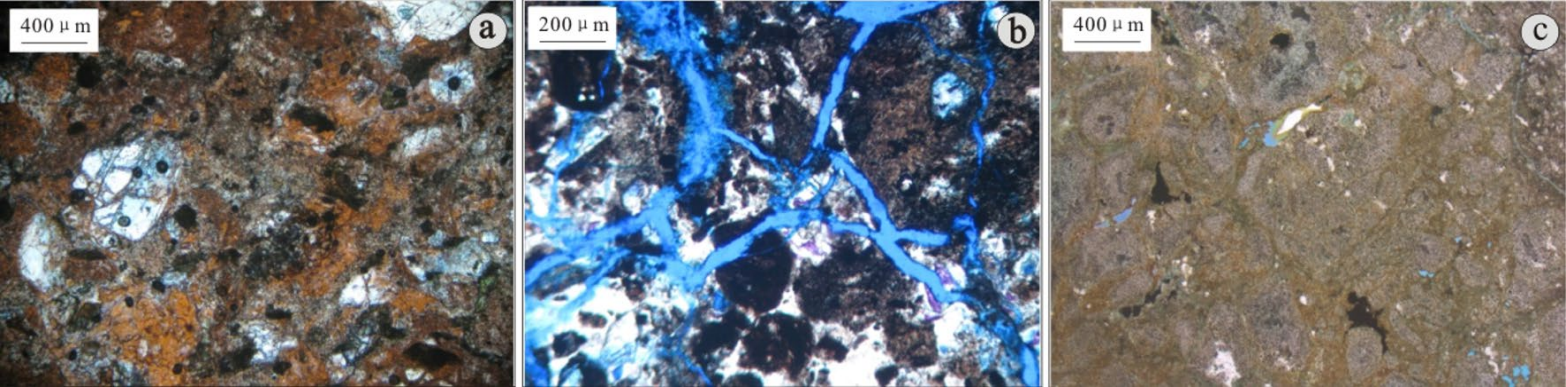
Characteristics of Mesozoic sedimentary volcaniclastics in the D oilfield of the Laizhouwan Sag. (**a**) Sedimentary tuff D-4 1219 m. (**b**) Sedimentary tuff D-4 1223 m. (**c**) Sedimentary volcanic breccia D-6 1491 m.

**Figure 5:**
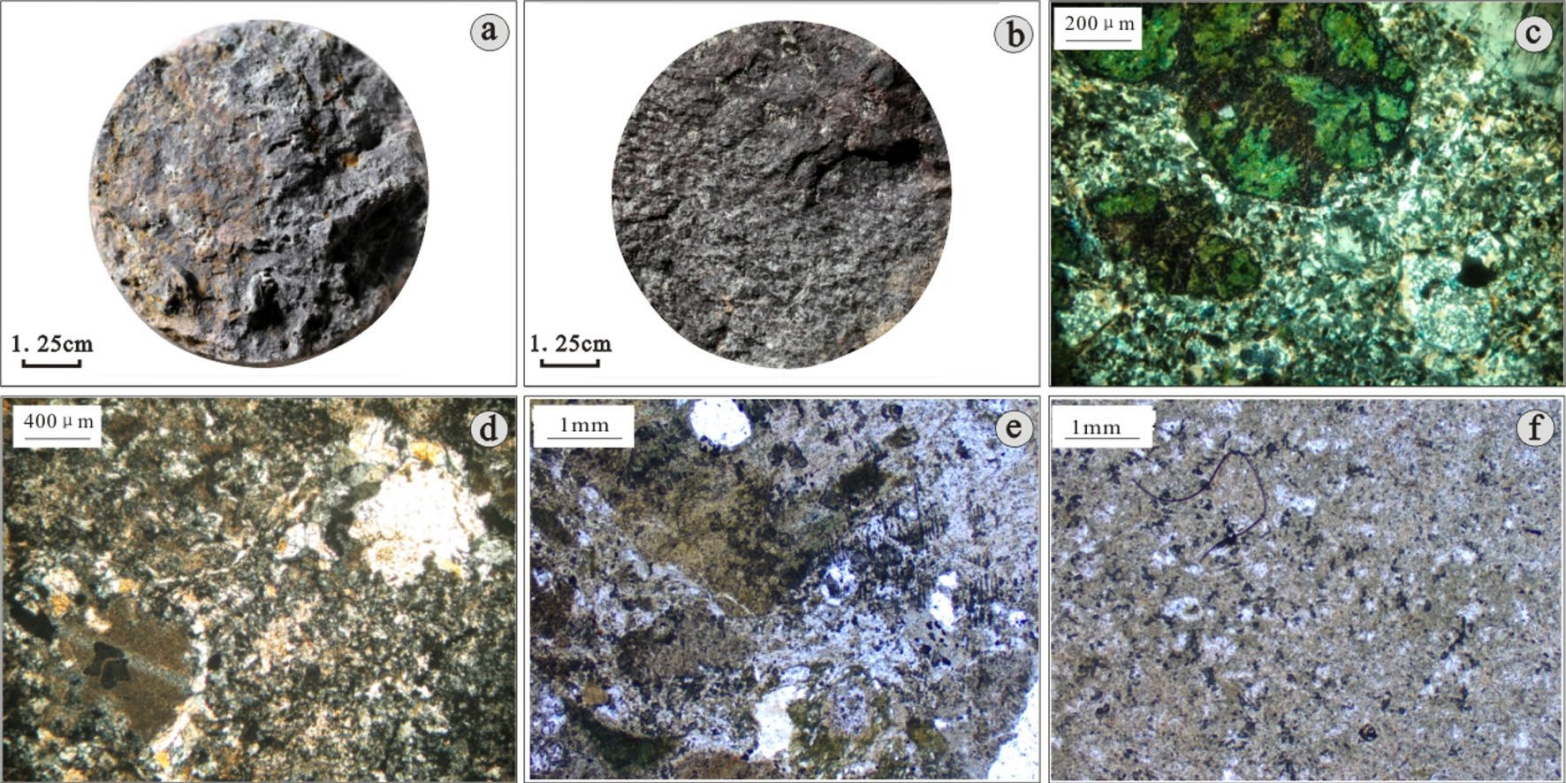
Characteristics of Mesozoic volcanic lava in the D oilfield of the Laizhouwan Sag. (**a**) Andesite D-4 1495 m porphyry structure. (**b**) Andesite D-6 1506 m massive structure. (**c**) Dacite D-7 1605 m intergranular structure. (**d**) Dacite D-9 1633 m glass crystal interwoven structure. (**e**) Rhyolite D-10 1775 m cryptocrystalline structure. (**f**) Rhyolite D-10 1733.5 m cryptocrystalline structure.

1. *Volcanic clastic rock*
  The tuff is mainly composed of fine volcanic ash and dust, contains a small amount of quartz, hornblende and biotite, with massive structure and tuff structure, is dense and hard, and has been nonuniformly altered (Fig. 3a,b,e). The volcanic breccia, with a range of colours, mainly grey, greyish green and reddish brown, is subangular and poorly sorted. The matrix is mainly composed of dense volcanic ash with fracture development and a volcanic breccia structure (Fig. 3c,d,f).
2. *Sedimentary pyroclastic rock*
  The sedimentary volcaniclastic rock is a transitional lithology between the volcaniclastic rock and the sedimentary rock formed under the dual transformation of volcanism and sedimentation. The sedimentary tuff is largely variegated, including mainly rock debris and volcanic dust. The rock debris includes primarily quartz and feldspar of medium and fine sand grades. Most of the rock debris has been altered, and some of the fragments are argillized. The matrix is mainly siliceous rock formed by volcanic dust under high temperatures and pressures and evenly distributed among rock cuttings, with moderate fracture and pore development and a tuff structure (Fig. 4a,b).The sedimentary volcanic clastic rocks developed in the study area are mainly sedimentary volcanic breccia and tuff. The sedimentary volcanic breccia is mainly variegated. The rock is mainly composed of volcanic breccia and mudstone around the breccia. The volcanic breccia has distinct edges and corners. The breccia is mainly composed of tuff rock blocks with poor pore development. The fractures are well developed, and the structure of the sedimentary volcanic breccia is also well developed (Fig. 4c).
3. *Volcanic lava*
  Volcanic lava is a kind of rock formed by the condensation and crystallization of magma from the weak part of the Earth's crust. The volcanic lava in the study area includes rhyolite, dacite and andesite. The andesite is mainly brownish grey and black/brown with a porphyritic structure. The phenocrysts are feldspar and plagioclase, and the matrix is fine plagioclase with an interwoven structure. The pores of the rock are poorly developed; the fractures include a small amount of chlorite and argillaceous filling, a small number of phenocryst dissolution pores exist, and chloritization and feldspar phenocryst kaolinization has occurred (Fig. 5a,b). The dacite is mainly grey, and the plagioclase and hornblende are mainly observed in phenocrysts. The plagioclase appears argillized. The matrix has a glass base interwoven structure. The plagioclase microcrystals are disorderly distributed in glassy areas. The observed cracks in these rocks are mostly filled by siliceous minerals and calcite; the rock is relatively dense, with a porphyritic texture (Fig. 5c,d). In the rhyolite, the quartz and feldspar are mainly observed in phenocrysts. In the later stage of phenocryst formation, the alteration was intense. The matrix has a cryptocrystalline structure, and fractures and cataclastic structures can also be observed (Fig. 5e,f).

### Recognition of lithology using a conventional log intersection chart

The lithology of volcanic rocks in the study area is complex, with neutral, basic and acidic rocks. In this paper, eleven wells in the study area were cored, core slices were made, and the natural gamma, neutron, acoustic time difference, resistivity, natural potential and density curves were calibrated with the determined lithology. At present, the intersection chart of the resistivity RD, ZDEN, CNCF and GR is mainly selected for detailed volcanic lithology identification.

**Figure 6:**
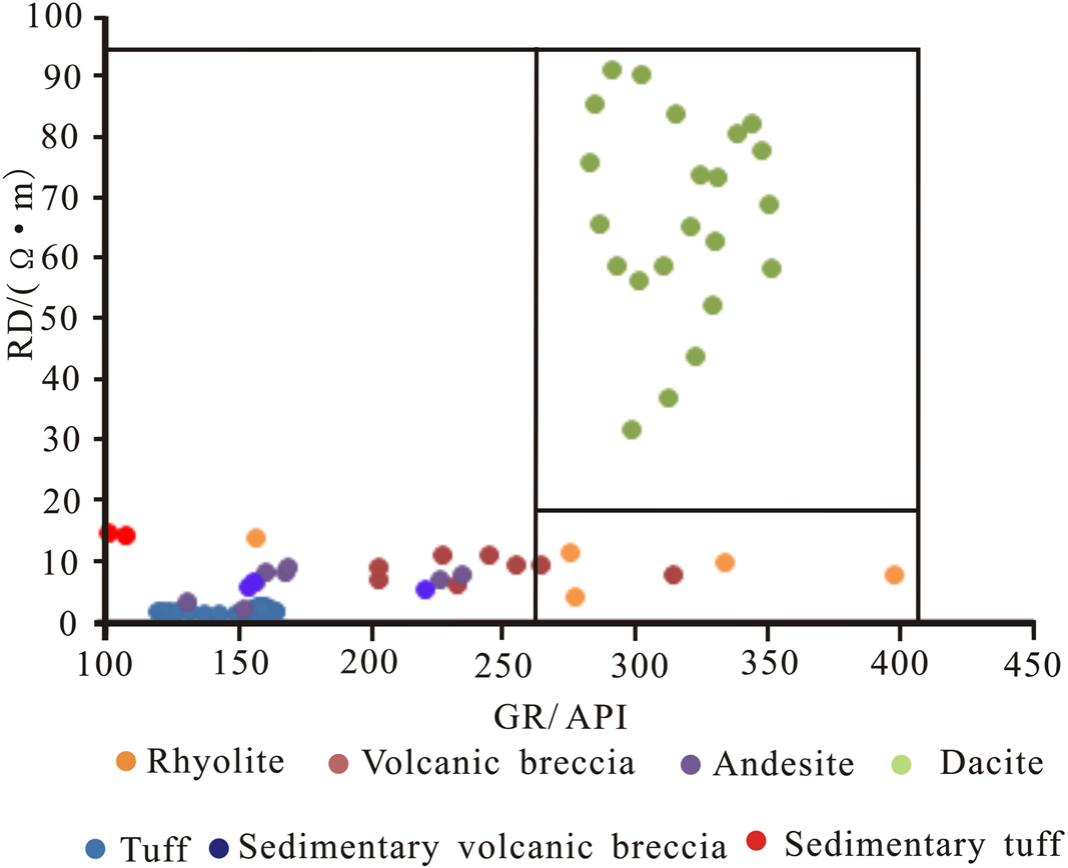
RD-GR crossplot of conventional logging curves of volcanic rocks in the study area.

It can be seen from the RD-GR crossplot (Fig. 6) that when RD < 20 Ω·m and GR < 270 API, the main lithologies identified are tuff, andesite, volcanic breccia, sedimentary tuff and sedimentary volcanic breccia. The RD value of tuff is less than 3 Ω·m, and the RD value of andesite and sedimentary breccia is in the range of 3–9 Ω·m. When GR > 250 API, the main lithology is dacite and tuff, and the RD value of dacite is generally greater than 30 Ω·m.

**Figure 7:**
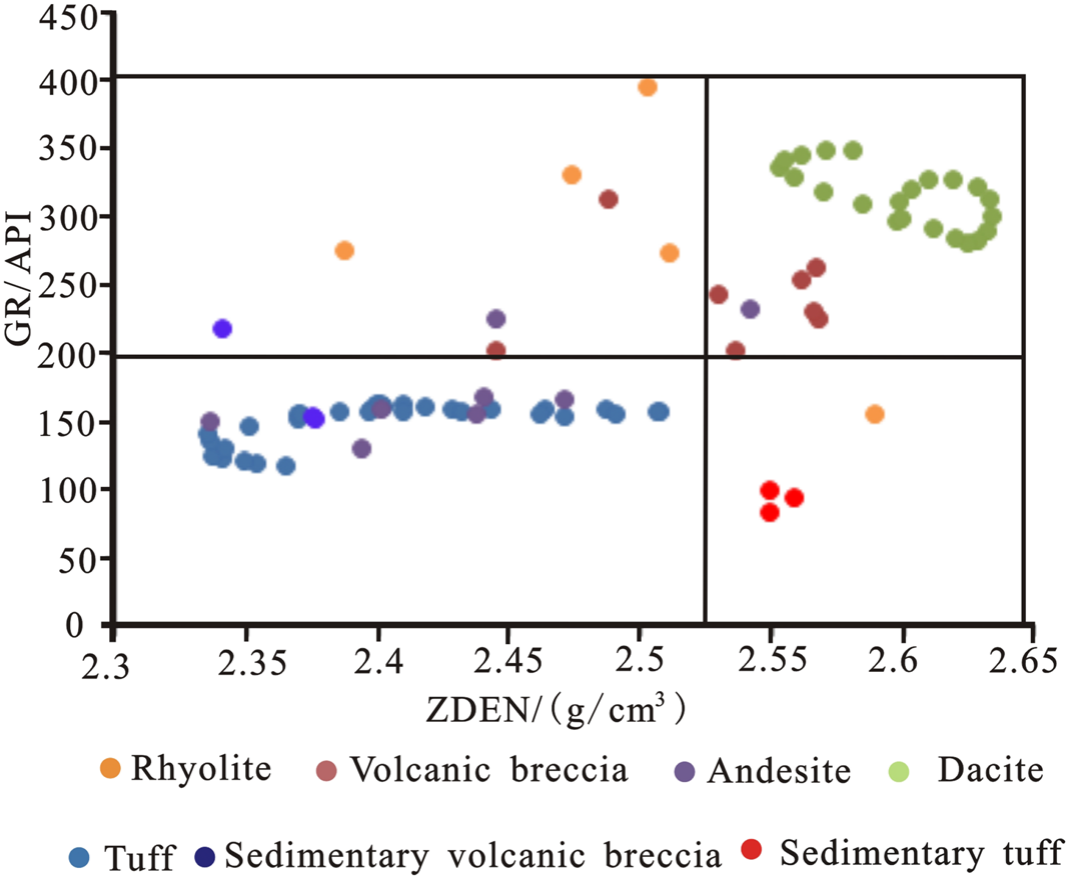
GR-ZDEN crossplot of conventional logging curves of volcanic rocks in the study area.

According to the GR-ZDEN intersection diagram (Fig. 7), when ZDEN < 2.53 (g/cm^3^) and GR < 200 API, the main lithologies identified are tuff, andesite and sedimentary volcanic breccia. When ZDEN < 2.53 (g/cm^3^) and 200 < GR < 400 API, the main lithologies identified are breccia bearing rhyolitic tuff and rhyolite. When ZDEN > 2.53 (g/cm^3^) and GR < 200 API, the identified lithology is tuff. When ZDEN > 2.53 (g/cm^3^) and GR > 200 API, volcanic breccia and dacite are identified.

**Figure 8:**
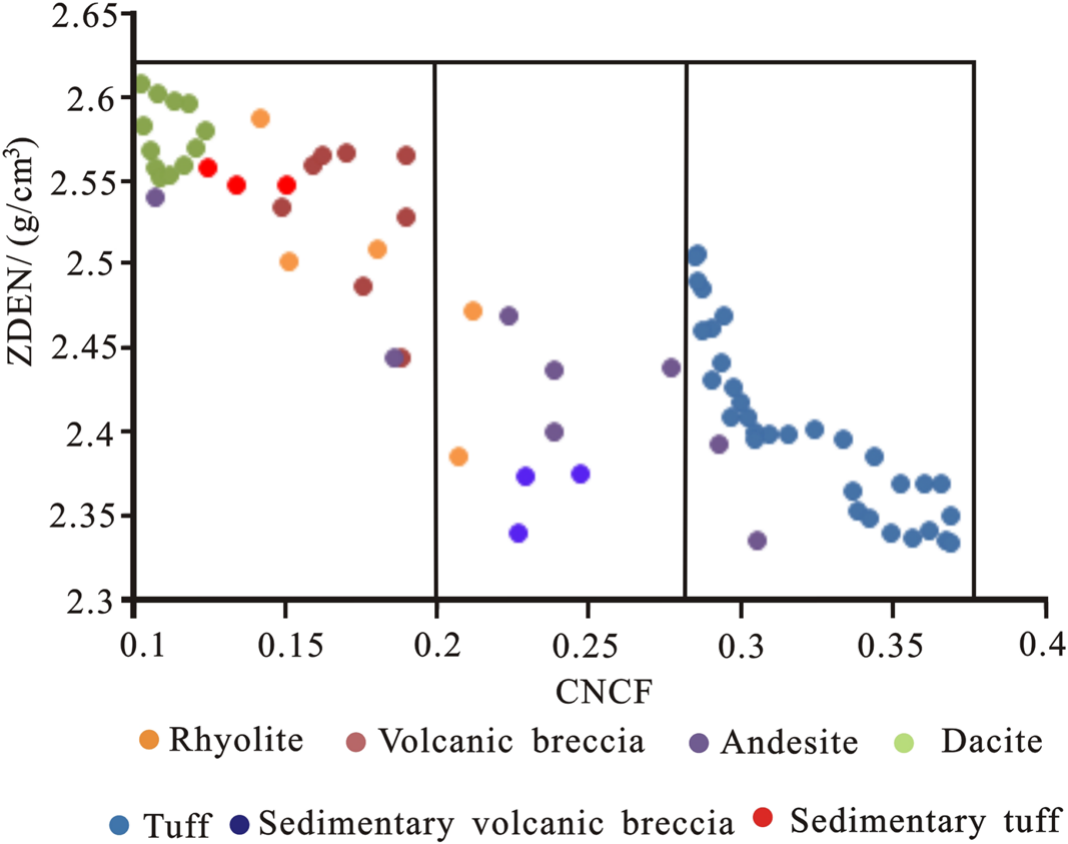
ZDEN-CNCF crossplot of conventional logging curves of volcanic rocks in the study area.

From the ZDEN-CNCF intersection map (Fig. 8), it can be seen that when CNCF > 0.258%, tuff is the main identified lithology. When CNCF < 0.258 and ZDEN < 2.46 (g/cm^3^), the main identified lithologies are breccia-bearing rhyolitic tuff, andesite and sedimentary volcanic breccia. When CNCF < 0.2% and ZDEN > 2.46 (g/cm^3^), the main identified lithologies are volcanic breccia, rhyolite, dacite and tuff.

### Curve characteristic method

Volcanic rocks of different lithologies have different logging response characteristics. The density curve, neutron curve and acoustic time difference curves in the study area are sensitive to the composition of the rock. For the conventional logging curves, the pyroclastic rocks are characterized by a low density, high density and high acoustic time difference, reflecting the high porosity and low resistivity of the reservoir. Among them, the logging response characteristics of volcanic breccia are obviously affected by its lithology. Specifically, the neutron density presents "zigzag double track" characteristics, there is a crossing phenomenon in the plot, and the deep lateral resistivity value is moderately high. The logging of tuff response characteristics is obvious, the neutron density has "smooth double track" characteristics, there is no crossing phenomenon in the plot, and the deep lateral resistivity is low. Volcanic lava is characterized by a high resistivity, certain "bayonet shape" in the plot, density close to the skeleton value, small neutron value, and low and straight acoustic transit time curves (Table 1).

**Table 1:** Lithology logging identification standard of the Mesozoic buried hill.

Rock logging classification	Response characteristics of conventional logging		
	RD (Ω·m)	ZDEN (g/cm^3^)	CNCF
Lava	> 15	> 2.65	< 0.15
Volcanic breccia	1–15	2.25–2.73	< 0.28
Tuff	< 10	< 2.57	0.08–0.4

### Imaging logging identification method

According to the change in the resistivity depth response of the borehole wall, FMI can directly and clearly observe the characteristics of rock structure, lithology and fractures and is used here to distinguish different volcanic rock lithology in the study area and improve the accuracy of volcanic rock lithology identification.

The volcanic lava is relatively dense and homogeneous on the whole, usually exhibiting a bright a blocky response and showing a high-resistivity in the FMI images, with dark stripes on either side of the high resistivity. In the FMI images, the andesite is mainly characterized by a massive and bright pattern, and dark arc-shaped bands are visible. The rhyolite is characterized by the combination of a layered bright pattern and a dark linear pattern. Dacite usually shows a combination of massive patterns and very fine dark stripes.

**Figure 9:**
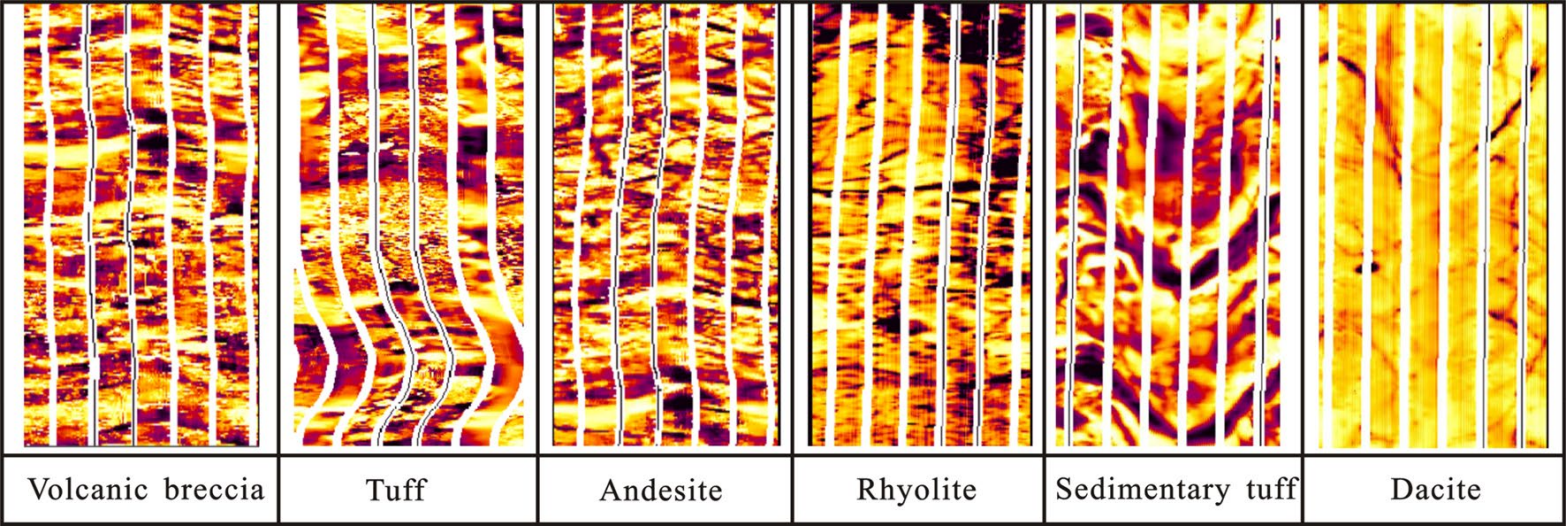
Imaging logging lithology identification chart.

Among the pyroclastic rocks, the volcanic breccia is mainly f volcanic breccia without a rounded structure. The interstitial materials are mainly volcanic dust and volcanic ash, which are the main rock types of volcanic explosive facies. The resistivity difference between volcanic breccia and volcanic ash is obvious, and the mode of an irregular combination of bright spot appears in the FMI images. Tuff is mainly composed of pyroclastic, crystalline, wavy and lithic material, with a layered or massive structure. Its resistivity is lower than that of the lava and pyroclastic rock. The volcanic breccia or gravel mixed locally in the tuff is bright, so the tuff exhibits a dark spotted pattern. The sedimentary tuff is characterized by alternating dark and bright bands in the FMI images, which reflect the general imaging logging characteristics of sedimentary rocks (Fig. 9).

### Identification of lithology by the decision tree method

**Figure 10:**
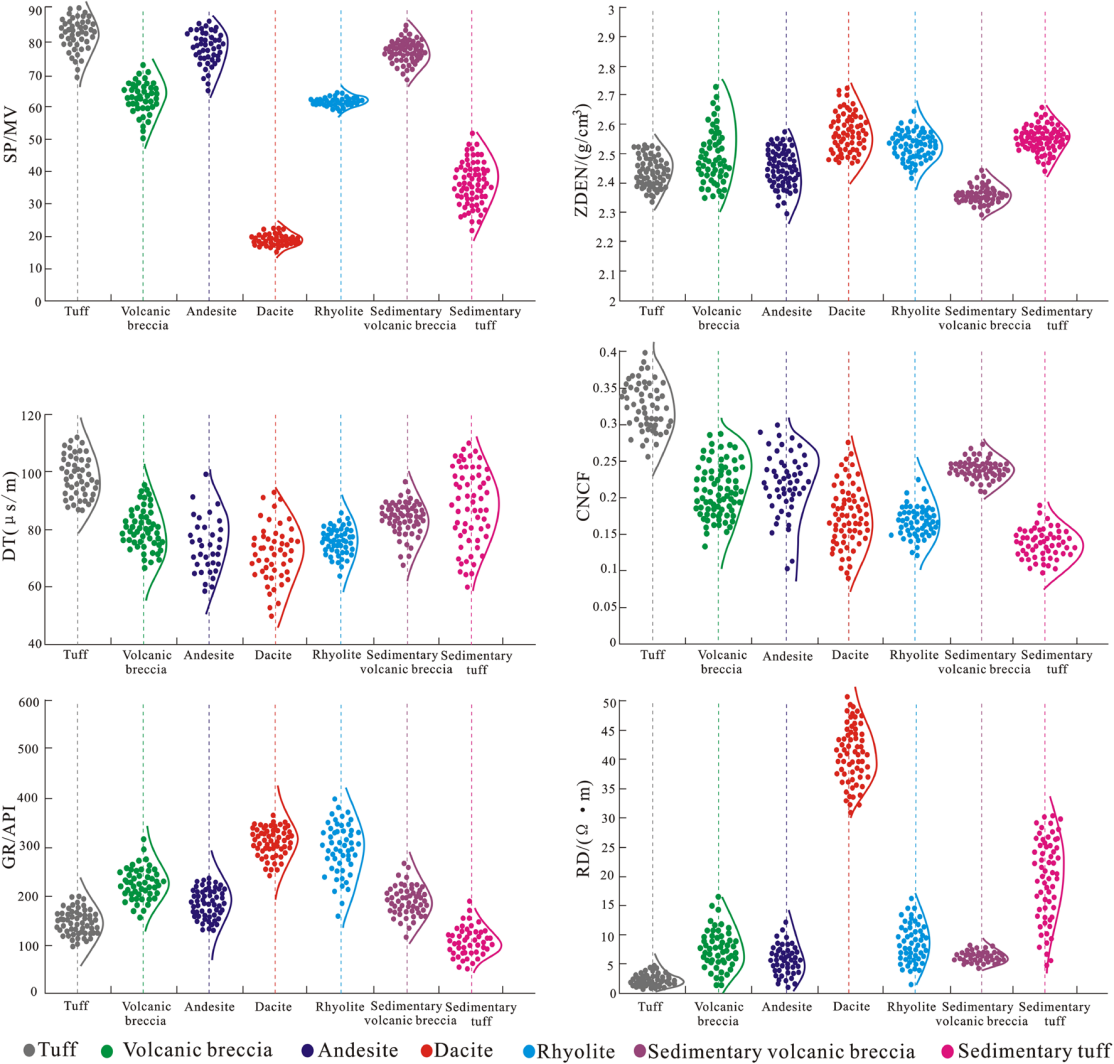
Probability density distributions of the well parameters.

The decision tree method can clearly describe the relationship between various logging curves and the lithology of volcanic rocks; thus, the optimization of logging parameters is very important in various lithology identification methods. The decision tree method can not only determine the adaptability of various lithology identification methods but also determine which kind of rock exhibits the best accuracy in the lithology identification. In general, the fewer logging parameters used for reference, the higher the lithological discrimination is. Usually, a set of specific logging parameters will be most sensitive to a certain rock response (Fig. 10). The decision tree method is a process of segmenting a large number of samples by recursive selection of optimal features. Based on the differences in radioactivity, porosity, density, acoustic velocity, conductivity and potential variation of various volcanic rocks, the logging response values corresponding to six logging curves (i.e., natural gamma, neutron, density, acoustic time difference, resistivity and spontaneous potential) of various volcanic rocks are taken as the total sample parameter set. The data set is divided into subsets by selecting sensitive curves, and then feature selection and partition are performed recursively until all subsets are correctly classified or there are no features available for segmentation to establish a decision tree and realize the recognition of volcanic rock lithology.

First, according to the logging data, six logging parameters are analysed in terms of their correlation to the dacite, andesite, rhyolite, tuff, volcanic breccia, sedimentary breccia and tuff in the study area, and the probability density of all the logging parameters of these seven volcanic rocks is projected into a map. A probability density distribution diagram of logging parameters can show the response sensitivity and approximate distribution range of various logging parameters to different types of volcanic rocks, and the approximate distribution range of logging response parameters corresponding to different lithologies can be observed. From the obtained probability density distribution diagram of the logging parameters, it can be seen that the CNCF curve is more sensitive to tuff, and many of the CNCF values of tuff are more than 0.25%, which can be clearly distinguished from other lithologies. However, other lithologies have little difference in terms of the values of the CNCF curves, which is difficult to distinguish. In addition, the GR values of dacite and rhyolite are relatively high, but the difference is not large, making it is difficult to distinguish them. The RD curve is relatively sensitive to dacite, remaining above 20 Ω·m, and the RD values of other lithologies are similar. Therefore, the RD curve can be used to identify dacite, but other curves are difficult to distinguish. The SP curves are sensitive to dacite and generally remain below 30 mV, but there is little difference between these three lithologies, making it is difficult to distinguish them. Therefore, the linear division of a single logging parameter cannot classify the lithology of complex volcanic rocks. Therefore, it is necessary to integrate multiple logging parameters to identify these lithologies.

**Figure 11:**
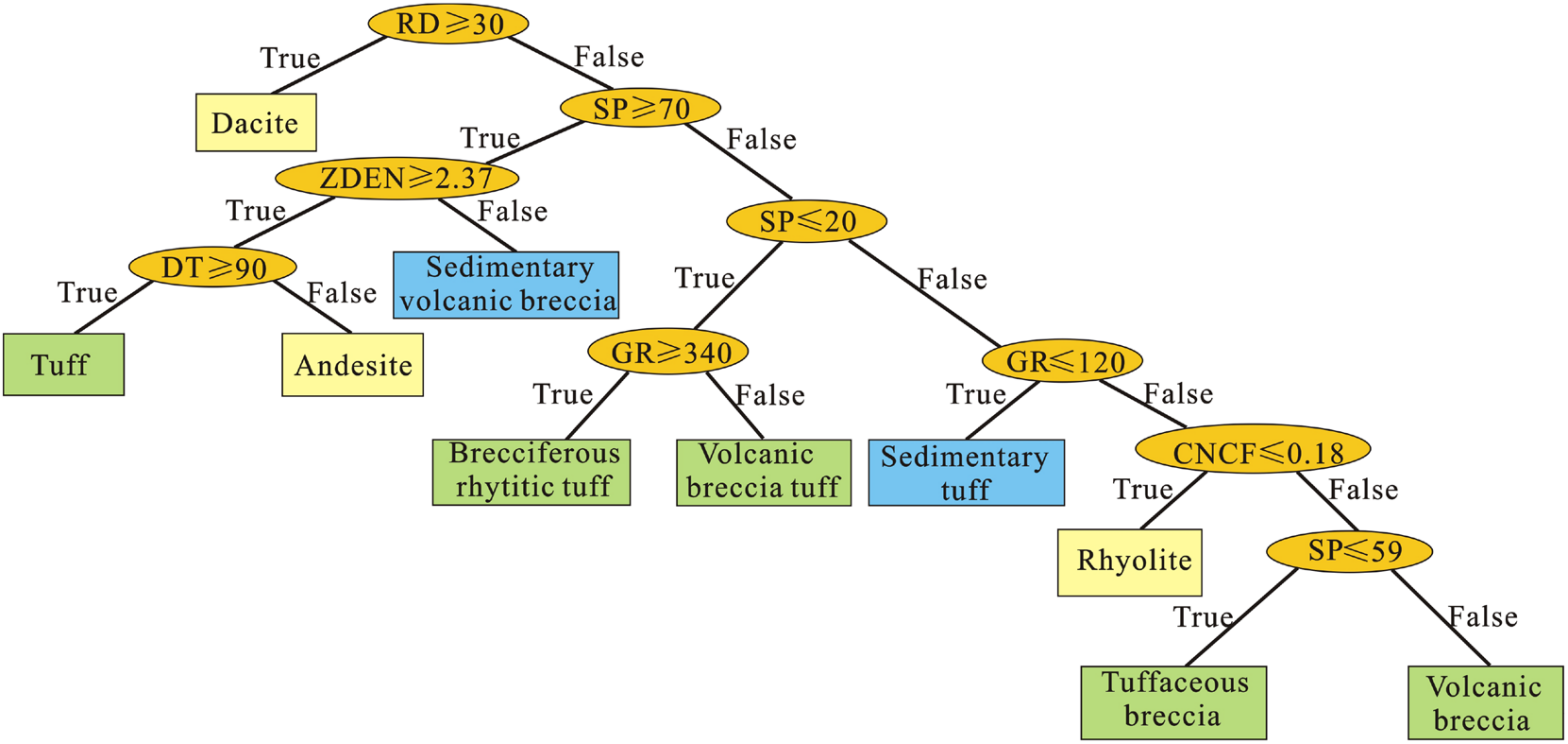
Lithology classifier based on the decision tree method.

To obtain the lithologic classification model of complex volcanic rocks, based on the probability density distribution characteristics of the logging parameters, the decision tree method is used to summarize all the logging parameter data in the study area, and logging parameter sample sets of different lithologies are established. For various lithologies, 215 logging parameter samples are randomly selected to obtain the sample parameter set. Based on the comprehensive analysis of the probability density distribution characteristics of the logging parameters, six logging parameters, namely, the acoustic (DT), natural gamma ray (GR), density (ZDEN), deep lateral resistivity (RD), compensated neutron (CNCF) and spontaneous potential (SP) parameters, are selected for lithology classification and identification. The required data set is obtained through the screening test of the sample set, and then a lithologic classifier that can identify the complex volcanic rock is gradually established according to the decision tree method (Fig. 11).

The lithology is segmented using the established lithology classifier of complex volcanic rocks, and the information entropy of each lithology type decreases with the layer screening. Taking RD = 30 Ω·m as the node, values greater than 30 Ω·m are divided into dacite, and the rest of the lithology is divided by the SP curve. When SP ≤ 20 MV and GR < 340 API, the lithology is volcanic breccia tuff; when SP ≤ 20 MV and GR ≥ 340 API, the lithology is breccia-bearing rhyolite tuff; when SP ≥ 70 MV and ZDEN < 2.37 g/cm^3^, the lithology is sedimentary volcanic breccia; when SP ≥ 70 MV, ZDEN ≥ 2.37 g/cm^3^ and DT ≥ 90 μs/m, the lithology is tuff; when SP ≥ 20 MV, ZDEN ≥ 2.37 g/cm^3^ and DT < 9 μs/m, the lithology is andesite; when SP is 20–70 MV and GR ≤ 120 API, the identified lithology is sedimentary tuff; when SP is 20–70 MV, GR > 120 API and CNCF ≤ 0.18, the identified lithology is rhyolite; when SP is 20–70 MV, GR > 120 API, CNCF > 0.18 and SP ≤ 59 MV, the identified lithology is tuffaceous volcanic breccia; and when SP is 20–70 MV, GR > 120 API, CNCF > 0.18 and SP > 59 MV, the identified lithology is tuffaceous volcanic breccia. The decision tree branch of each layer can divide the logging curve data. The model composed of multiple logging parameter branches to identify the volcanic rock lithology by the decision tree method can more clearly reflect the logging response characteristics of volcanic rock to improve the recognition accuracy of the volcanic rock lithology. Therefore, the cutoff value table of the volcanic rock lithology logging identification is summarized in this study (Table 2).

**Table 2:** Cutoff values of lithology identification based on the decision tree method.

Lithology	CNCF	DT	GR	RD	ZDEN	SP
Andesite	0.11–0.3	59.5–90.58	130.38–233.82	2.02–9.06	2.37–2.54	72.06–84.56
Dacite	0.091–0.28	56.82–80.41	282.07–350.83	27.46–91.22	2.49–2.65	18.06–20.51
Rhyolite	0.14–0.18	68.7–80.73	156.12–397.63	4.11–14.36	2.39–2.59	60.16–62.46
Tuff	0.28–0.37	90–110.06	118.57–163.67	1.46–2.71	2.37–2.51	80.33–88.11
Volcanic breccia	0.18–0.28	68.76–89.15	173.59–263.28	1.48–11.77	2.32–2.73	59–66.01
Tuffaceous breccia	0.18–0.34	71.47–107.63	201.3–268.55	1.8–8.34	2.36–2.46	55.45–59.92
Breccia-bearing rhyolitic tuff	0.2–0.23	67.32–94.31	340–485.39	7.01–11.92	2.37–2.53	9.08–11.03
Volcanic breccia tuff	0.21–0.23	73.71–82.82	300.85–340	7.47–9.3	2.4–2.49	9.08–10.45
Sedimentary volcanic breccia	0.23–0.25	83.53–89.82	152.69–219.77	5.27–6.59	2.34–2.37	76.29–79.68
Sedimentary tuff	0.11–0.16	62.82–105.31	85.19–121	4.768–31.85	2.52–2.58	2.5–49.25

## Discussion

### Comparison and application of different lithology identification methods

**Figure 12:**
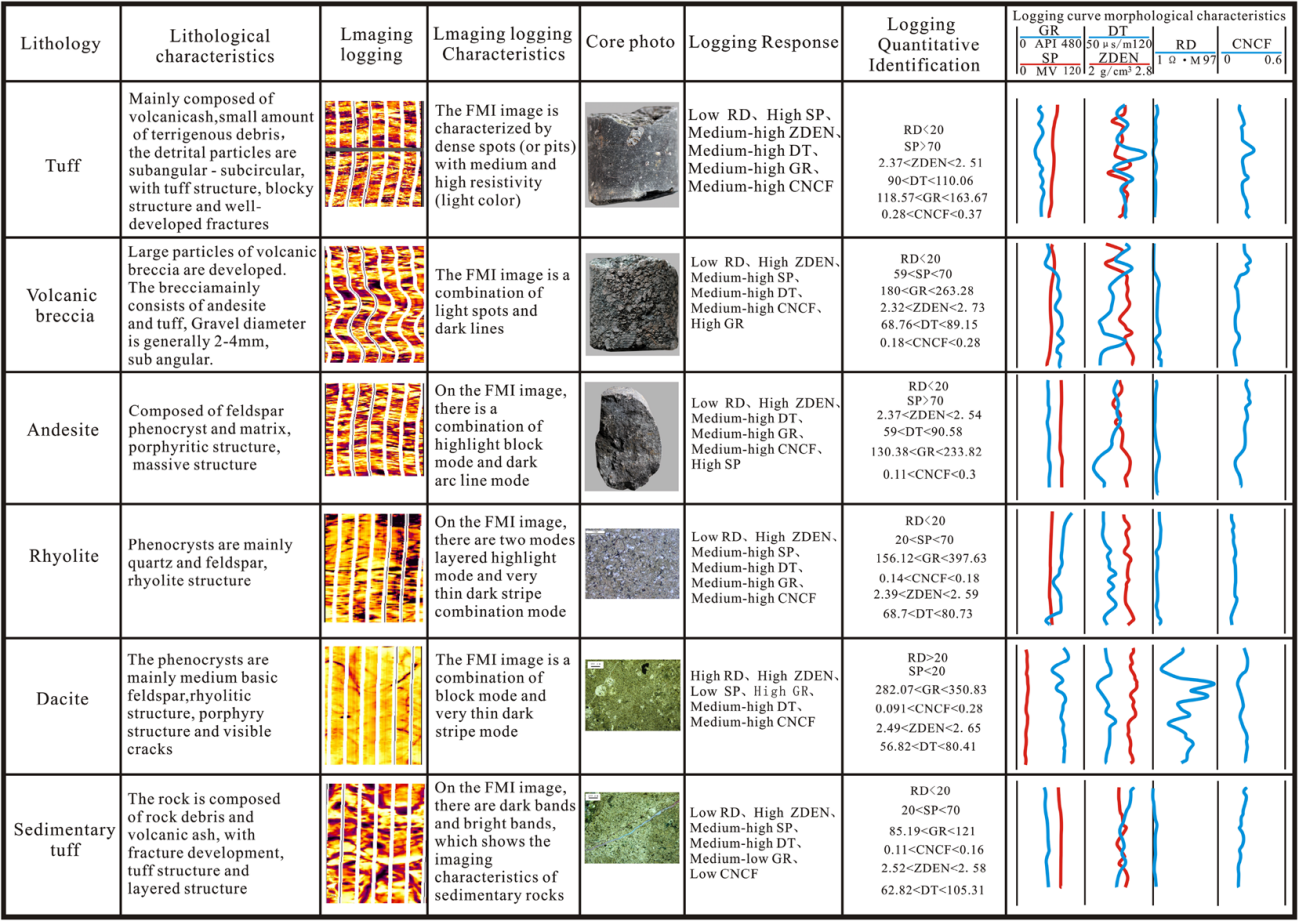
Logging curve, core slice identification and imaging logging identification chart of Mesozoic volcanic rocks in the Laizhouwan Sag.

Based on the decision tree method to identify lithology and using the constraints of the FMI results, curve shape and crossplot discrimination results, a lithology identification chart is established with core and thin-section photographs (Fig. 12).

**Figure 13:**
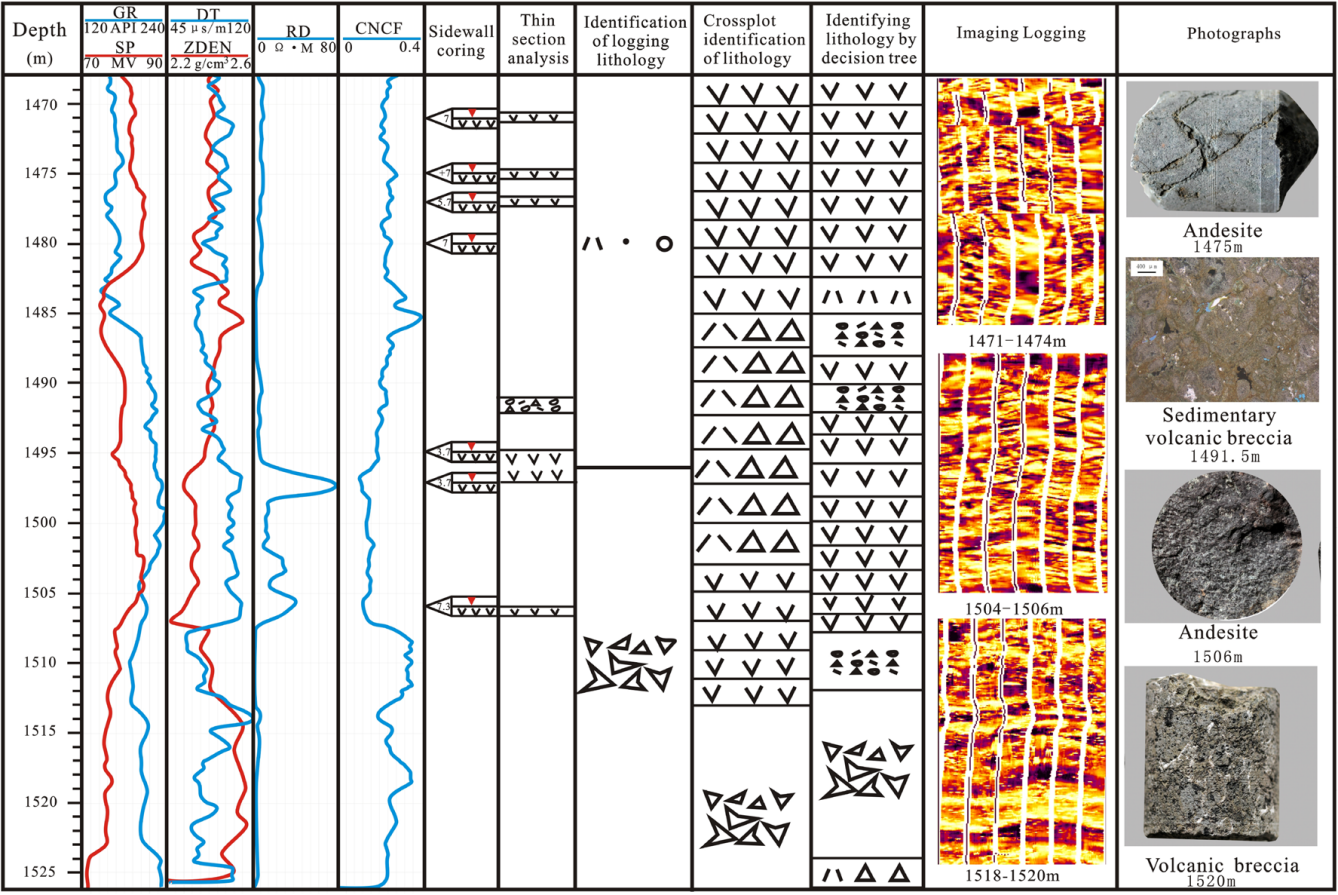
Comparison of the original lithology and corrected lithology of the D-6 well.

Through the comparative analysis of back judgement, it is shown that there are errors in the identification of rock composition by different methods, in which the lithology identification error of the intersection map is smaller than that of the curve shape method, while the error of lithology identification by the decision tree method is smaller than that of the crossplot method. This shows that the error of a single curve in lithology identification is greater than that of lithology identification with multiple curves. Imaging logging has a high accuracy but also a high cost, and most old wells lack imaging logging data. Therefore, imaging logging and core photographs can be used as references for lithology identification and correction to improve the accuracy of volcanic rock lithology identification. The decision tree method is used to identify the lithology of each well in the study area. Taking well D-6 as an example, the lithology identification results are compared with the core photographs. In the 1468–1485 m well section, when RD < 30 Ω·m, SP > 70 MV, ZDEN > 2.37 g/cm^3^, and DT < 90 μs/m, the original logging lithology identification suggested tuffaceous sand conglomerate. However, it can be seen from the decision tree method that the well section is mainly andesite, and it can also be seen from the core photographs that the section is andesite. In the 1485–1492 m interval, when RD < 30 Ω·m, SP > 70 MV, and ZDEN < 2.37 g/cm^3^, the lithology identified by logging is tuffaceous sand conglomerate, while sedimentary volcanic breccia is mainly developed in this interval according to the decision tree method. In the 1495–1508 well section, when RD < 30 Ω·m, SP > 70 MV, ZDEN > 2.37 g/cm^3^, and DT < 90 μs/m, the lithology identified by logging is volcanic breccia, but the decision tree method shows that andesite is mainly developed in this section. It can be seen from the borehole coring photographs and core slice photographs that the core identified by the decision tree method is more in line with the actual situation (Fig. 13). The results show that the application of the decision tree method to the lithology identification of the mixed rock in the study area is generally good, and its accuracy rate exceeds 82%.

### Influence of volcanic lithology on oil bearing properties

**Figure 14:**
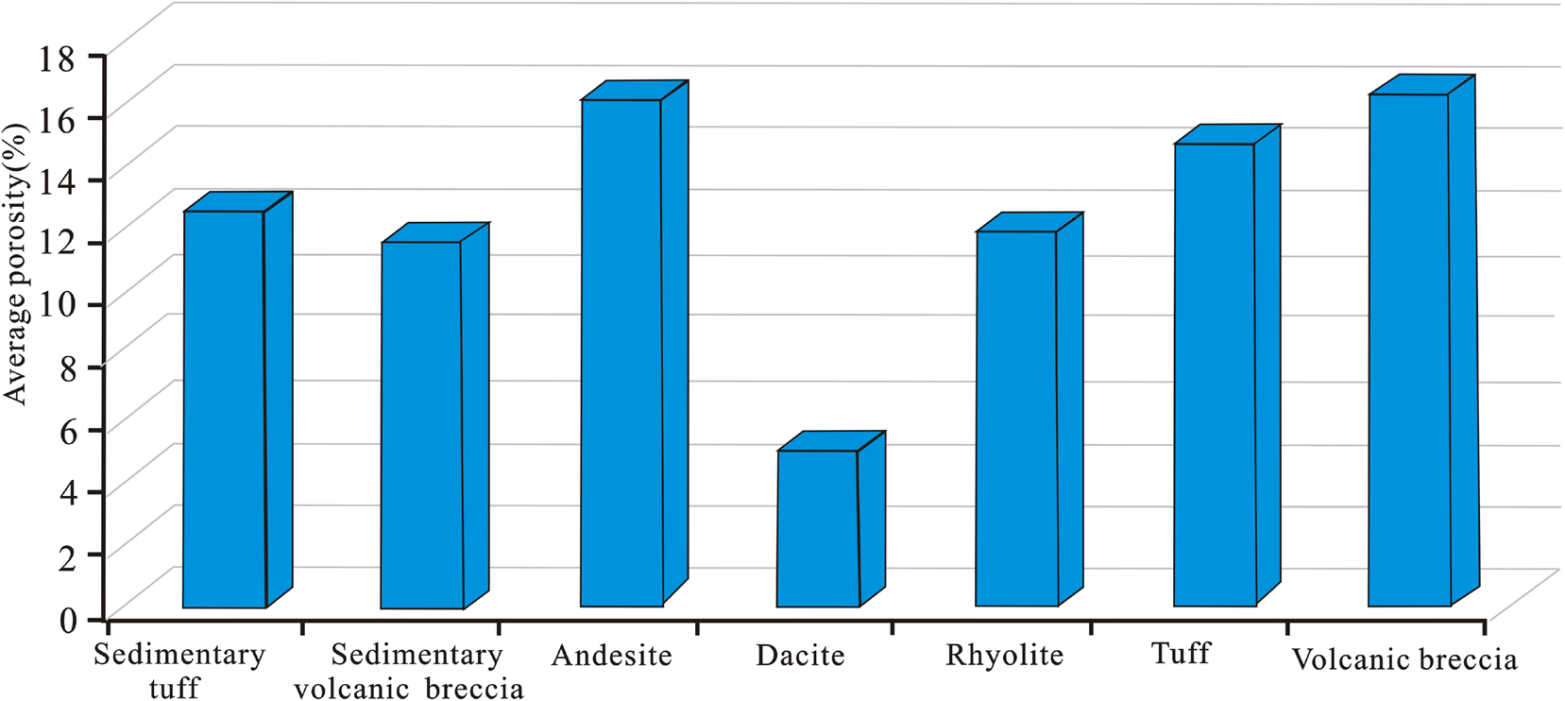
The average porosity of different lithologies.

Through the comprehensive analysis of the volcanic rock lithology identification, logging oil-bearing level analysis, reservoir space type identification and porosity lower limit, oil–gas reservoir comprehensive identification of the test wells in this area (Table 3) is carried out, and the results are in good agreement with the oil test results. According to the statistical results of the physical properties of different lithologies, the physical properties of different types of volcanic rocks are clearly different. The lithologies with the most favourable physical properties are tuff, volcanic breccia and andesite, which have relatively high porosities; the average porosity of andesite is 16.02%, that of volcanic breccia is 16.185%, and that of tuff is 14.7% (Fig. 14). According to the relationship between the oil-bearing properties and lithology, andesite has the most favourable oil-bearing properties, followed by tuff and volcanic breccia. The logging display level of volcanic rock cuttings in the oil layer (oil spotting) can be used as an important threshold to identify oil and gas reservoirs.

**Table 3:** Comprehensive identification table of the Mesozoic volcanic rock oil layer.

Well	Oil grade	Lithology	Reservoir space	Porosity	Comprehensive discrimination	Test results (daily oil production/m^3^)
D-9	Oil spotting	Tuff	Fracture	12.5	Oil layer	16.9
D-6	Oil spotting	Volcanic breccia	Fracture, dissolution pore	16.8	Poor reservoir	9.5
D-6	Oil spotting	Andesite	Fracture	14.3	Poor reservoir	18.5
D-6	Oil spotting	Volcanic breccia	Fracture, dissolution pore	18.2	Oil layer	2.8
D-6	Oil spotting	Tuff	Fracture	12.1	Oil layer	3.2
D-6	Fluorescence	Andesite	Fracture, dissolution pore	15.6	Oil layer	1.7

In conclusion, the three types of volcanic rock lithologies, andesite, tuff and volcanic breccia, easily form oil and gas reservoirs in this area. Identifying these three lithologies is an important basis for field identification of oil and gas reservoirs. The lithology of volcanic rock is one of the important factors that affect the oil-bearing properties of volcanic reservoirs.

## Conclusion

1. Based on the analysis of the characteristics of the lithology parameters, the characteristics of the logging parameters of different lithologies are clarified. Six logging parameters that are sensitive to lithology, including the natural gamma ray, density, acoustic, compensated neutron and deep lateral resistivity parameters, are selected. Then, the decision tree method is used to distinguish them sequentially. Finally, the mode and technical method of mixed lithology identification are established, with a combination of the conventional logging crossplot method, the decision tree method, imaging logging and core calibration.
2. The volcanic rocks in the study area are mainly composed of volcanic lava (andesite, dacite, and rhyolite), pyroclastic rock (tuff and volcanic breccia) and sedimentary pyroclastic rock (sedimentary volcanic breccia and tuff). Compared with other lithology identification methods, the lithology identified by this decision tree method is more in line with the actual situation. According to the relationship between the oil-bearing properties and lithology, andesite has the most favourable oil-bearing properties, followed by tuff and volcanic breccia. The logging display level of volcanic rock cuttings in the oil layer (oil spotting) can be used as an important threshold to identify oil and gas reservoirs.
